# Complete Genome Sequence of Mannheimia varigena Isolated from Bovine Milk

**DOI:** 10.1128/MRA.01377-18

**Published:** 2019-02-21

**Authors:** Matthew S. McCabe, Gaelle Esnault, Gerard Murray, Bernadette Earley, Paul Cormican

**Affiliations:** a Teagasc Grange, Animal & Grassland Research and Innovation Centre, Dunsany, County Meath, Ireland; b Department of Agriculture, Food and Marine, Sligo Regional Veterinary Laboratory, Doonally, Sligo, Ireland; University of California, Riverside

## Abstract

Mannheimia varigena is a pathogen of cattle that has been isolated from diseased lung and udder. There are currently complete genome sequences for 4 M. varigena isolates, all from lungs of cattle in the United States. We report a complete genome sequence of M. varigena isolated from bovine milk in Ireland.

## Abstract (precis)

Mannheimia varigena is a pathogen of cattle that has been isolated from diseased lung and udder. There are currently complete genome sequences for 4 M. varigena isolates, all from lungs of cattle in the United States.

## ANNOUNCEMENT

Mannheimia varigena is a Gram-negative bacillus that has been isolated from diseased bovine lungs (1, 2), bovine udder and spleen where association with disease was not known (3), and bovine meninges from a fatal case of meningitis (4). Little is known about the pathogenicity-associated genes of this bacterial species. There are currently complete closed genome sequences for 4 M. varigena isolates, which were all isolated from lung necropsies taken from U.S. cattle with shipping fever (1). We used a combination of long-read Nanopore sequencing and short-read Illumina sequencing to generate a complete closed circular genome of a bacterium that was isolated in Ireland from milk from a cow with clinical mastitis. A charcoal swab of this milk isolate (received from the Sligo Regional Veterinary Laboratory) was used to inoculate a Columbia agar plate (supplemented with 5% defibrinated sheep’s blood), resulting in colonies of identical morphology. A single colony was picked from the plate and grown in brain heart infusion (BHI) broth in the dark at 37°C for 20 h without shaking.

DNA was extracted from the bacterial culture with a QIAamp cador pathogen minikit (Qiagen, UK) according to the manufacturer’s instructions. A Nanopore sequencing library was generated from this DNA with the 1D^2^ sequencing kit (R9.5) (Oxford Nanopore Technologies, Oxford, UK) following the “1D^2^ sequencing of genomic DNA (with SQK-LSK308) protocol” that was available on the Oxford Nanopore Technologies website in June 2017, except that the DNA fragmentation step was omitted. Long-read sequencing of the Nanopore library was conducted on a MinION Mk1B Nanopore sequencer (Oxford Nanopore Technologies) on a MAP107 (R9.5.1) flow cell generating 203,938 reads, with an average read length of 3.88 kb and a maximum read length of 395.5 kb.

A TruSeq DNA PCR-free low throughput library prep kit (Illumina, San Diego, CA) was used to generate an Illumina library from the same bacterial DNA sample that was used for the Nanopore library preparation. This Illumina library was sequenced on an Illumina MiSeq platform with a 500-cycle reagent kit to generate 391,572 paired-end (2 × 250 bp) reads.

Assembly of the combined Illumina and Nanopore FastQ sequence files was performed with Unicycler 4.0 (5) and Circlator 1.5.5 (6) with the default parameter settings. This resulted in a single contiguous circularized sequence of 2,167,239 bp. The top hits of a BLAST search of the entire 2,167,239 bp assembly against the nonredundant nucleotide (nr/nt) bacterial NCBI database were the M. varigena complete genomes (97 to 98% identity, 94 to 96% coverage), the *Mannheimia* sp. USDA-ARS-USMARC-1261 complete genome (84% identity, 90% coverage), the *Pasteurellaceae* bacterium 12565 chromosome (72% identity, 83% coverage), the M. haemolytica strain 11935 chromosome (72% identity, 83% coverage), the M. haemolytica strain 193 chromosome complete genome (79% identity, 83% coverage), and the M. haemolytica strain 187 chromosome complete genome (78% identity, 83% coverage).

Annotation of the M. varigena Teagasc 1 assembly with the NCBI Prokaryotic Genome Annotation Pipeline (PGAP) (7, 8) showed 1,950 protein-coding genes, 60 tRNAs, 4 noncoding RNAs, 8 5S rRNA, 6 16S rRNA, 6 18S rRNA, 35 pseudogenes, and 2 CRISPR arrays. This is the first report of a complete genome sequence of M. varigena isolated from bovine milk.

### Data availability.

The GenBank nucleotide sequence accession number for Mannheimia varigena strain Teagasc 1 is CP030062. Raw sequences are available in the Sequence Read Archive (BioProject number, PRJNA416915; SRA sample, SRS4180578; SRA study, SRP174186).
